# Allergic Bronchopulmonary Aspergillosis: A Contemporary Narrative Review

**DOI:** 10.2174/0118743064513774260904230554

**Published:** 2026-10-13

**Authors:** Turki Almalki

**Affiliations:** 1 Department of Internal Medicine, College of Medicine, Prince Sattam bin Abdulaziz University, Al-Kharj, Saudi Arabia

**Keywords:** Allergic bronchopulmonary aspergillosis, *Aspergillus fumigatus*, Asthma, Cystic fibrosis, Bronchiectasis, Biologic therapy, Antifungal therapy, ISHAM criteria

## Abstract

Allergic Bronchopulmonary Aspergillosis (ABPA) is an immune-mediated airway disease caused by hypersensitivity to *Aspergillus fumigatus* in genetically and immunologically susceptible people, most often those with asthma or Cystic Fibrosis (CF). Persistent luminal fungus, impaired mucociliary clearance, and exaggerated type 2 immunity interact to produce mucus plugging, eosinophilic inflammation, bronchiectasis, and, in advanced disease, respiratory failure. The 2024 International Society for Human and Animal Mycology (ISHAM) criteria require *A. fumigatus* sensitization and total immunoglobulin E (IgE) ≥500 IU/mL, together with supportive immunologic or imaging evidence. Sensitization without active ABPA is a related but distinct state and is not routinely treated.

This narrative review integrates evidence on epidemiology, immunopathogenesis, diagnosis, clinical classification, radiology, differential diagnosis, complications, and treatment. Prednisolone or itraconazole monotherapy is appropriate for most acute disease; combined therapy is generally reserved for recurrent exacerbations. Therapeutic Drug Monitoring (TDM) is important during azole treatment. Biologics directed against IgE, interleukin (IL)-5/IL-5 receptor α, or IL-4 receptor α are increasingly used for treatment-dependent disease, but the evidence is mainly observational. Cystic Fibrosis Transmembrane Conductance Regulator (CFTR) modulators may reduce airway fungal burden but create clinically important cytochrome P450 3A4 (CYP3A4) interactions with azoles. Response and relapse should be judged against a patient-specific stable IgE baseline together with symptoms and imaging.

## 1 INTRODUCTION

Allergic Bronchopulmonary Aspergillosis (ABPA) develops when *Aspergillus fumigatus* persists within airway mucus in a genetically and immunologically susceptible host. Impaired mucociliary clearance contributes to persistence but is not, by itself, a sufficient explanation: epithelial dysfunction, antigen presentation, and amplified type 2 immunity shape the clinical syndrome. Recurrent mucus obstruction and inflammation can lead to bronchiectasis and fixed airflow limitation, whereas the fungus ordinarily remains within the airway lumen rather than invading the lung parenchyma [1, 2].

The presentation overlaps with uncontrolled asthma, infection, eosinophilic lung disease, and non-ABPA bron-chiectasis. This review therefore emphasizes operational decisions: whom to test, how to apply the revised ISHAM criteria, when not to treat sensitization alone, how to choose anti-inflammatory *versus* antifungal therapy, and how to interpret serial IgE in asthma and CF [1].

## 2 LITERATURE SEARCH METHODOLOGY

A structured literature search of PubMed/MEDLINE, EMBASE, and the Cochrane Library was undertaken for publications from January 2000 through July 27, 2026. Search concepts included “allergic bronchopulmonary aspergillosis,” “allergic bronchopulmonary mycosis,” “*Aspergillus fumigatus*,” “fungal sensitization,” “asthma,” “cystic fibrosis,” “bronchiectasis,” “omalizumab,” “mepolizumab,” “benralizumab,” “dupilumab,” “itraconazole,” “basophil activation,” “CFTR modulator,” and “ISHAM.” Reference lists of key guidelines and reviews and ClinicalTrials.gov were also examined.

Selection was purposive, as appropriate for a narrative review. Priority was given to contemporary clinical practice guidelines, randomized controlled trials, systematic reviews and meta-analyses, prospective diagnostic studies, clinically informative cohorts, and foundational reports retained for historical context. Publications were excluded when they were duplicative, not directly relevant, superseded by stronger evidence, or lacked sufficient clinical detail. No formal study-level risk-of-bias instrument or certainty grading was applied. Accordingly, study design, size, follow-up, endpoint heterogeneity, and major conflicts of interest were considered qualitatively and are stated where they materially affect interpretation. The final reference list was checked for relevance, citation order, bibliographic accuracy, and retraction notices. Where several sources provided equivalent support, preference was given to the most methodologically informative citation, and redundant citations from the same author group were avoided.

## 3 EPIDEMIOLOGY AND RISK FACTORS

### 3.1 Clinical Predispositions

Asthma and CF are the best-established predisposing conditions. ABPA also occurs in non-CF bronchiectasis and, less often, in chronic obstructive pulmonary disease or in a patient with a compatible clinico-radiologic presentation without a previously recognized predisposing disorder. Reported prevalence varies widely with the population and diagnostic criteria; tertiary cohorts are enriched for severe disease and should not be extrapolated to unselected asthma populations. Atopy is best considered a host phenotype that increases the probability of fungal sensitization, not a separate mechanistic category [1, 3].

### 3.2 Mechanisms of Susceptibility

Mechanistic susceptibility includes mucus retention, epithelial barrier injury, impaired mucociliary clearance, and altered innate and adaptive immune signaling. CFTR dysfunction changes airway-surface physiology; Human Leukocyte Antigen (HLA) associations and variants in cytokine or pattern-recognition pathways have also been reported. These associations are biologically plausible but are not sufficiently discriminative for routine genetic testing [2-4].

### 3.3 Immunopathogenesis and Etiology

#### 3.3.1 The Causative Organism and Airway Persistence

*Aspergillus fumigatus* is the principal trigger of ABPA. Other *Aspergillus species* and filamentous fungi, including *Curvularia*, *Helminthosporium*, and *Fusarium* species, may cause Allergic Bronchopulmonary Mycosis (ABPM). Small conidia reach distal airways, germinate in retained mucus, and expose fungal cell-wall components and proteases. In an immunocompetent host, mucociliary transport, epithelial antimicrobial defenses, macrophages, and neutrophils ordinarily limit persistence [2, 4].

#### 3.3.2 Innate Immune Defects and Genetic Susceptibility

Airway epithelium and antigen-presenting cells recognize fungal ligands through pattern-recognition receptors, including Dectin-1 and Toll-like receptors (TLR-2 and TLR-4); complement-associated pathways contribute in parallel but are not themselves pattern-recognition receptors. Surfactant proteins, mannose-binding lectin, and phagocyte function participate in opsonization and clearance. These pathways interact with HLA-linked antigen presentation and other host factors rather than constituting a single causative defect [2, 3].

#### 3.3.3 Type 2 Immune Amplification

Fungal products stimulate epithelial release of IL-25, IL-33, and Thymic Stromal Lymphopoietin (TSLP). These alarmins activate Dendritic Cells (DCs) and type 2 innate lymphoid cells (ILC2s); DC antigen presentation and cytokine signals then promote polarization of naive CD4+ T cells toward a type 2 T-helper (Th2) phenotype. IL-4 and IL-13 promote IgE class switching and mucus hypersecretion, whereas IL-5 supports eosinophil maturation and survival. This sequence is a synthesis of convergent mechanistic evidence rather than a single linear pathway demonstrated in every patient [2-4].

#### 3.3.4 Simplified Cascade

- Airway persistence and germination expose fungal ligands and proteases [2, 4].
- Dectin-1/TLR signaling and complement-associated pathways activate epithelial and innate responses [2, 3].
- IL-25, IL-33, and TSLP activate DCs and ILC2s [2, 4].
- DCs and cytokines promote Th2 polarization; IL-4/IL-13 drive IgE and mucus, while IL-5 drives eosinophilia [2-4].
- Mucus obstruction, eosinophilic injury, and repeated inflammation produce bronchiectasis and advanced ABPA [1, 3].

This framework links pathogenesis to phenotype-directed therapy: omalizumab reduces free IgE; mepolizumab and benralizumab interrupt the IL-5/eosinophil axis; and dupilumab blocks IL-4 receptor α, inhibiting IL-4 and IL-13 signaling. Phenotype matching is rational, but no head-to-head ABPA trial has established a superior biologic [5, 6].

### 3.5 Clinical Manifestations

Patients may present with difficult-to-control asthma, wheeze, cough, sputum, dyspnea, recurrent exacerbations, brown mucus plugs, blood eosinophilia, or transient/migratory opacities. Mucus plugging may cause lobar collapse. In CF, these findings overlap with the underlying disease, so disproportionate deterioration and serial change are particularly important [1, 7].

*Aspergillus* sensitization, defined here as *A. fumigatus*-specific IgE ≥0.35 kUA/L or a positive immediate skin test, does not equal ABPA. The revised ISHAM guidance does not recommend routine treatment for asymptomatic ABPA or sensitization alone. Observation is reasonable unless there is mucus plugging, bronchiectasis progression, or worsening asthma; in such patients, review with chest radiography and total IgE every 3–6 months can be individualized [1].

### 3.6 Diagnostic Approach and Classification

#### 3.6.1 Revised 2024 ISHAM Criteria

The 2024 ISHAM framework requires a predisposing condition (asthma, CF, chronic obstructive pulmonary disease, bronchiectasis) or a compatible clinico-radiologic presentation. Both *A. fumigatus* sensitization and total IgE ≥500 IU/mL are essential, with at least two of three supportive components: *A. fumigatus*-specific IgG, blood eosinophils ≥500 cells/μL (current or historical), and suggestive thoracic imaging. High-Attenuation Mucus (HAM) is pathognomonic and can establish ABPA even if other components are incomplete. A total IgE below 500 IU/mL may exceptionally be accepted when all other criteria are met, particularly after glucocorticoid exposure or at age extremes [1].

*A. fumigatus*-specific IgE is preferred over a skin test when available. Recombinant Asp f1, f2, and f4 IgE can support difficult cases but are not universally accessible. Galactomannan is not recommended for diagnosing ABPA because it is designed primarily for invasive aspergillosis and lacks suitable performance in this setting. Table **1** translates the criteria into their diagnostic roles [1, 8].

### 3.7 Clinical Classification: ISHAM 2024 and Historical Staging

Rosenberg–Patterson staging is retained only for historical and educational context; it should not be applied concurrently to make modern treatment decisions. ISHAM 2024 uses operational, dynamic states that better reflect relapsing disease. Importantly, older 35%-response and 100%-relapse IgE thresholds have been replaced by a ≥20% decline as one response component and a ≥50% rise from the stable new baseline as one required component of exacerbation. Table **2** summarizes the transition [1, 9, 10].

### 3.8 Radiology, Bronchoscopy, and Physiologic Assessment

Thin-section chest CT is the preferred baseline imaging study. Findings include central or peripheral bronchiectasis, mucus impaction, HAM, centrilobular nodules, tree-in-bud opacities, mosaic attenuation, air trapping, consolidation, and atelectasis. HAM is highly specific and indicates a greater mucus burden. Chest radiography is useful for interval assessment but cannot reliably exclude bronchiectasis [1, 11].

Pulmonary function commonly shows airflow limitation, measured by reduced forced expiratory volume in 1 second (FEV_1_). Serial FEV_1_ provides an objective measure of airway impairment but is not specific for ABPA activity. Bronchoscopy is not routine; it is useful for refractory lobar collapse, suspected alternative infection, or a diagnostically uncertain mucus-plugging syndrome [1, 4].

### 3.9 Differential Diagnosis

The most common mimic is Severe Asthma with Fungal Sensitization (SAFS), in which fungal sensitization is present without the immunologic and radiologic constellation required for ABPA. Other important differential diagnoses include eosinophilic pneumonia, hypereosinophilic syndrome, non-ABPA bronchiectasis, Chronic Pulmonary Aspergillosis (CPA), Eosinophilic Granulomatosis With Polyangiitis (EGPA), bronchocentric granulomatosis, and ABPM due to other fungi. Table **3** highlights practical discriminators [1, 12].

### 3.10 Complications and Long-term Outcomes

Repeated mucus obstruction and inflammation may lead to progressive bronchiectasis, fixed airflow limitation, recurrent bacterial infection, lobar collapse, hemoptysis, pulmonary hypertension, and chronic respiratory failure. CPA is a distinct superimposed diagnosis; its risk is most relevant in patients with advanced structural lung disease and prolonged or cumulative immunosuppression, not after a short glucocorticoid course in isolation [1, 12].

The course is heterogeneous. Approximately half of patients may experience an exacerbation after stopping acute therapy, whereas 10%–25% develop treatment-dependent disease. Reliable pooled modality-specific relapse estimates are unavailable because trials use different entry criteria, response definitions, and follow-up windows. In CF, a 2024 pediatric and adolescent cohort linked ABPA and *Aspergillus* colonization with adverse long-term outcomes, but modern CFTR-modulator-era trajectories require prospective confirmation [1, 13].

### 3.11 Treatment Principles

Treatment aims to control active inflammation, reduce fungal burden when appropriate, relieve mucus obstruction, prevent exacerbations and structural progression, and minimize toxicity. Background asthma or CF therapy and airway clearance should be optimized. The 2024 ISHAM guideline does not recommend routine systemic therapy for asymptomatic ABPA [1].

### 3.12 Therapeutic Evolution and Randomized Trial Evidence

The 2016 Infectious Diseases Society of America (IDSA) guidance emphasized glucocorticoids, with itraconazole as an adjunct or steroid-sparing therapy. ISHAM 2024 accepts either oral prednisolone or itraconazole monotherapy for acute ABPA and recommends their combination primarily for recurrent exacerbations [1, 12].

In the unblinded 2018 itraconazole-versus-prednisolone trial (n=131), the 6-week composite response favored prednisolone (100% *vs* 88%; *P*=0.007), while IgE decline, lung function, and 1- and 2-year exacerbations were similar; glucocorticoid adverse effects were more frequent. An independent systematic review of antifungal therapy identified four randomized trials and 34 observational studies across asthma and CF; oral azoles generally improved clinical, immunologic, and radiologic outcomes, but the certainty of evidence was low or very low because of limited controlled data and heterogeneity. In the 2022 trial (n=191), 1-year exacerbation was numerically lower with prednisolone plus itraconazole than with prednisolone alone (20.6% *vs* 33.0%; *P*=0.054), but the prespecified comparison was not statistically significant [14-16].

The two contemporary comparative RCTs were single-center studies. They used heterogeneous composite responses, treatment-failure definitions, and follow-up horizons, were not powered for modest differences in relapse, and provided limited external validation. The evidence therefore supports more than one reasonable first-line option rather than a universal ranking. Independent placebo-controlled trials of itraconazole also demonstrated clinical or immunologic benefit in treatment-dependent or stable ABPA, supporting a steroid-sparing and anti-inflammatory effect [14, 16-20].

### 3.13 Biologic Therapy: Evidence and Phenotype Matching

The 2024 meta-analysis included 86 reports and 346 patients, but only 16 omalizumab studies were suitable for pooled analysis, and most evidence for other agents came from individual patient data in case reports or small series. Omalizumab was associated with 2.29 fewer exacerbations per year (95% CI, 1.26–3.32), a 10.91-mg/day reduction in oral corticosteroid dose, and a 10.09-percentage-point improvement in FEV_1_. These uncontrolled estimates are susceptible to selection and publication bias and do not establish superiority over another biologic [5].

Phenotype can guide an individualized off-label choice: omalizumab for an allergic/high-IgE phenotype within the locally licensed dosing table; mepolizumab or benralizumab for eosinophil-predominant disease and mucus plugging; and dupilumab for broader IL-4/IL-13-driven type 2 disease or when IgE exceeds the omalizumab table. A 29-patient retrospective anti-IL-5/IL-5Rα series evaluated 32 weeks before and after treatment and reported fewer exacerbations, lower corticosteroid doses, better lung function, and mucus-plug improvement in 82%, despite no significant total-IgE change. This is clinically encouraging but not comparative efficacy evidence [5, 6]. Biologic switching after inadequate response or intolerance is increasingly reported, but the available evidence remains largely descriptive and case-based and does not establish an optimal treatment sequence [18].

A substantial proportion of ABPA patients have total IgE above a regional omalizumab dosing table, but no robust ABPA population estimate can be given because eligibility varies by jurisdiction, body weight, prior therapy, and cohort selection. Severe-asthma literature describes off-table omalizumab use above 1500 IU/mL, but it does not validate a high-dose ABPA regimen. Very high IgE (>2000 IU/mL) does not alter the diagnostic approach or mandate omalizumab; confirm ABPA using standard criteria, treat acute disease conventionally, and choose a licensed phenotype-appropriate biologic when treatment dependence persists [1, 19].

### 3.14 Contemporary Clinical Practice

Prednisolone is generally favored when rapid control is required because of hypoxemia, extensive opacities, marked symptoms, or severe mucus plugging. Itraconazole is reasonable for selected non-severe acute disease or when glucocorticoid toxicity is a major concern. Combination prednisolone–itraconazole should not be routine for every new diagnosis; its clearest role is in recurrent exacerbations. The explicit decision points are summarized in Fig. (**1**), and treatment regimens, outcome data, and monitoring considerations are summarized in Table **4** [1, 14, 16].

For itraconazole, guideline-based TDM should be obtained at approximately 2 weeks and 3 months after a formulation or dose change, and when clinical failure, toxicity, or a new interaction is suspected. A trough ≥0.5 mg/L is generally targeted; voriconazole trough ≥1 mg/L is used when that agent is selected. If a level is low, first verify adherence, formulation-specific food/acid instructions, interacting medicines, and the timing of the sample; then adjust the dose or switch the agent, with repeat TDM after the change. Blind dose escalation is unsafe [1, 21].

Where TDM is not routinely available, no validated alternative dosing algorithm reproduces its safety. A pragmatic pathway is standard guideline dosing, careful medication reconciliation, use of a formulation with predictable administration, baseline and serial liver tests, symptom/electrocardiographic toxicity surveillance when indicated, and referral or intermittent sample shipment for TDM when response is inadequate or an important interaction exists. If reliable exposure cannot be established, prolonged empiric dose escalation should be avoided [1, 21].

### 3.15 Monitoring, Response, Remission, and Relapse

Response should be assessed after 8–12 weeks using symptoms, chest imaging, and total IgE. ISHAM defines response at 8 weeks as ≥50% symptom improvement plus either major radiologic improvement or a ≥20% total-IgE decline. The lowest value achieved during clinical stability becomes the patient-specific “new baseline”; normalization is neither expected nor a treatment target [1].

An ABPA exacerbation requires sustained clinical worsening for at least 14 days or radiologic worsening, together with total IgE ≥50% above the stable new baseline, after excluding asthma exacerbation, bacterial infection, and bronchiectasis exacerbation. Thus, IgE alone can be falsely positive because of biologic or assay variability and falsely negative after glucocorticoid exposure or when a stable baseline was never established. *A. fumigatus*-specific IgE and IgG are not recommended as serial response biomarkers and should not be used to adjudicate a borderline total-IgE fluctuation in isolation [1].

These cutoffs must be interpreted as components of composite clinical states, not as isolated laboratory pass/fail rules. A patient with ≥50% symptomatic improvement and major radiologic clearing can meet the response definition even when the total-IgE fall is <20%; conversely, a ≥20% IgE fall without the required symptomatic improvement is insufficient. Similarly, a single ≥50% rise should prompt confirmation when it is discordant with the clinical picture, but it establishes an ABPA exacerbation only when the required clinical or radiologic worsening is also present, and competing causes have been excluded. Whenever feasible, serial values should be obtained with the same assay and laboratory [1].

During the first year after treatment, review every 3–6 months is reasonable; thereafter, 6–12-month intervals can be individualized. Targeted imaging is appropriate when symptoms, IgE, or lung function deteriorate; routine annual CT is unnecessary in stable disease. Table **5** distinguishes guideline recommendations from pragmatic toxicity surveillance [1].

### 3.16 Special Population: Cystic Fibrosis

The diagnostic tenets of ISHAM 2024 apply to CF: *A. fumigatus* sensitization and total IgE ≥500 IU/mL are essential in most patients, with supportive evidence. The historic Cystic Fibrosis Foundation criteria used >1000 IU/mL for “classic” ABPA and >500 IU/mL for a minimal case, with repeat testing when IgE was 200–500 IU/mL; they should not be described as a uniformly lower threshold. In current practice, the ISHAM framework should be applied while giving greater weight to serial change and alternative explanations because baseline bronchiectasis, mucus plugging, infection, and atopy overlap. Bacterial colonization should not be presented as a general direct cause of high IgE [1, 7].

CFTR modulators improve CFTR protein function and mucociliary clearance. Elexacaftor/Tezacaftor/Ivacaftor (ETI) has been associated with a marked reduction in *A. fumigatus* detection, and 2026 translational data suggest partial restoration of an epithelial interferon response. These observations support a disease-modifying hypothesis but do not establish that ETI prevents or treats active ABPA [13, 22, 23].

Drug interactions are bidirectional. Lumacaftor is a strong CYP3A4 inducer and may lower azole exposure; azoles inhibit CYP3A4 and can raise ivacaftor-containing modulator exposure. The correct response is not automatic itraconazole escalation or an unmonitored switch to voriconazole. Review the exact modulator and azole label, formulation, adherence, and co-medications; use TDM and apply recommended modulator dose adjustments. If adequate azole exposure cannot be achieved or tolerated, seek CF/pharmacy expertise and select an alternative rather than escalating blindly [1, 7].

### 3.17 Emerging Biomarkers in CF

A CF biomarker meta-analysis identified promising signals for recombinant allergen tests, stimulated basophil CD203c expression, and advanced imaging, but most estimates came from single, small studies. A prospective cohort of 67 patients contained only 6 ABPA cases; an optimized extract-BAT cutoff yielded 100% sensitivity and 77.6% specificity, with an Asp f4 signal. These thresholds are not ready for routine adoption [24, 25].

Operationally, BAT requires prompt processing of fresh blood, flow cytometry, controlled allergen concentrations, validated gating, and laboratory-specific cutoffs. Between-study differences in extract/component reagents and activation markers limit transferability. These requirements, small validation cohorts, and limited access in community laboratories are major barriers. Recombinant Asp f1/f2/f4 IgE may be used when available; galactomannan is not recommended for ABPA, and IgG4 has no validated diagnostic or monitoring threshold [1, 24, 25].

### 3.18 Unresolved Questions and Future Directions

First-line comparative evidence remains geographically concentrated and endpoint-heterogeneous. The PICA phase 3 trial (NCT06174922) compares prednisolone, itraconazole, and their combination; its registry was listed as recruiting when accessed on July 27, 2026, although registry status can lag site activity. A multicenter observational biologic study (NCT07362693) was listed as not yet recruiting, and the randomized phase 2 dupilumab study LIBERTY ABPA AIRED (NCT04442269) was listed as completed. Peer-reviewed reports and confirmatory trials remain necessary before these studies change practice [26-28].

Biomarker validation should prospectively prespecify thresholds, sample handling, analytical platforms, and clinically meaningful outcomes. Resource-stratified designs are essential: centralized BAT or TDM may be feasible through hub-and-spoke laboratories, whereas serial clinical assessment, total IgE, spirometry, radiography, liver testing, and medication reconciliation are more scalable. Trial protocols should measure cost, travel, sample stability, and implementation failure as well as biological performance [24].

Transient receptor potential vanilloid 1 (TRPV1) is a plausible neuroimmune modifier of allergic airways. In a chronic ovalbumin (OVA) murine asthma model, capsazepine or TRPV1 small-interfering RNA reduced airway hyperresponsiveness and inflammation, type 2 cytokines (IL-4, IL-5, and IL-13), epithelial alarmins (TSLP, IL-33, and IL-25), goblet-cell hyperplasia, smooth-muscle thickening, and collagen deposition. A separate OVA model with PM2.5/formaldehyde co-exposure linked capsazepine to lower pro-inflammatory neuropeptide and oxidative-stress signals. In the 2026 OVA study, however, the principal intervention was the CB2 agonist JWH133; capsazepine was used as a TRPV1 antagonist to probe mechanism rather than as an independently developed ABPA therapy [29-31].

The translational boundary is important: none of these studies used *Aspergillus* sensitization or challenge, measured fungal persistence or ABPA-specific mucus plugging, enrolled humans with ABPA, or established capsazepine pharmacokinetics, dosing, selectivity, and safety. Claims that capsazepine corrects metabolic or autophagic dysfunction in other experimental systems therefore cannot be extrapolated to ABPA. A rational research sequence would first confirm TRPV1 dysregulation in ABPA airway samples and *Aspergillus*-driven models, then test effects on fungal burden, mucus pathology, type 2 inflammation, and remodeling before toxicology and early-phase human studies. At present, TRPV1 blockade is appropriately identified as a hypothesis-generating future direction, not a treatment recommendation [29-31].

Future ABPA trials should harmonize the 2024 ISHAM response and exacerbation definitions, report exacerbation rate, FEV_1_, oral corticosteroid exposure, imaging response, quality of life, toxicity, and cost, and extend follow-up beyond 12 months [1].

## CONCLUSION

ABPA is a treatable consequence of persistent airway fungus and dysregulated type 2 immunity in a susceptible host. The 2024 ISHAM framework improves diagnostic consistency and replaces older response and relapse thresholds. Prednisolone and itraconazole are evidence-supported options for acute disease, whereas recurrent or treatment-dependent ABPA requires careful attention to azole exposure, cumulative glucocorticoid toxicity, and phenotype-directed biologic therapy [1, 5, 14].

The central practical principle is longitudinal integration: symptoms, imaging, lung function, and total IgE must be interpreted together against a stable patient-specific baseline. Prospective multicenter studies with standardized endpoints and resource-aware implementation plans are now needed to determine comparative treatment effectiveness and validate emerging biomarkers [1, 24].

## Figures and Tables

**Fig. (1):**
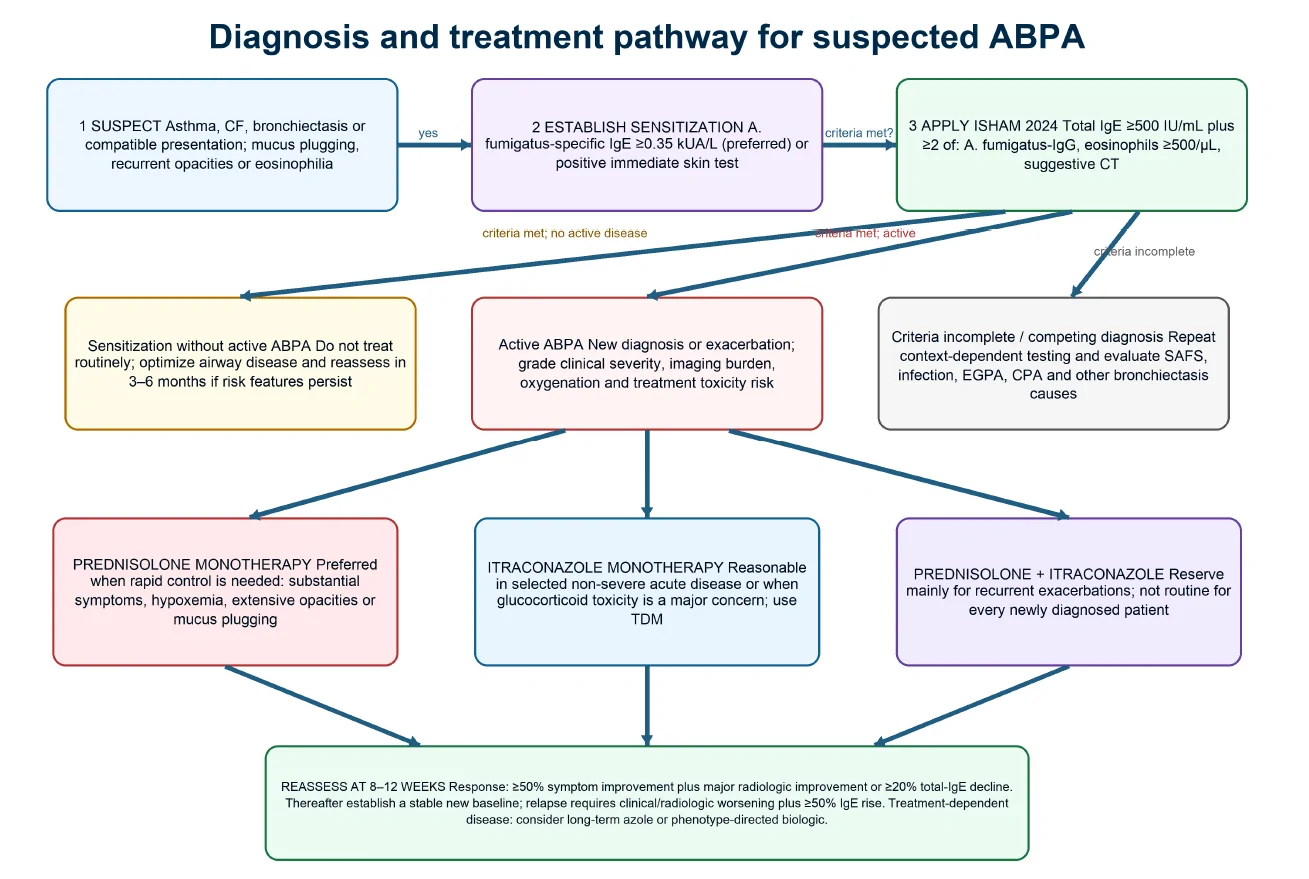
Practical stepwise approach to diagnosis and treatment of allergic bronchopulmonary aspergillosis. Legend: Active ABPA requires the 2024 ISHAM diagnostic constellation and clinico-radiologic activity. Severity, speed of control, contraindications, and recurrence determine selection of prednisolone, itraconazole, or combination therapy. TDM is required for azole exposure assessment [1].

**Table 1:** Revised 2024 ISHAM diagnostic criteria for allergic bronchopulmonary aspergillosis.

**Diagnostic Component**	**Requirement**	**Interpretation/role**
Clinical context	Predisposing condition or compatible clinico-radiologic presentation	Entry condition; ABPA is not limited to asthma or CF.
Essential criterion 1	*A. fumigatus*-specific IgE ≥0.35 kUA/L; immediate skin test if specific IgE is unavailable	Mandatory evidence of sensitization.
Essential criterion 2	Total IgE ≥500 IU/mL	Mandatory in most patients; a lower value may rarely be accepted if all other features are convincing.
Supportive criteria	At least 2 of 3: *A. fumigatus*-specific IgG; blood eosinophils ≥500 cells/μL; suggestive imaging	Additional evidence that distinguishes ABPA from sensitization alone.
Imaging	Bronchiectasis, mucus plugging, or other compatible opacities on thin-section CT	Defines structural burden and radiologic subtype.
Pathognomonic feature	High-attenuation mucus	Can confirm ABPA even when fewer additional criteria are present.
Key 2024 change	Total-IgE threshold reduced from 1000 to 500 IU/mL	Improves sensitivity while preserving the requirement for sensitization plus supportive evidence.

**Table 2:** ISHAM 2024 clinical states and historical Rosenberg–Patterson analogues.

**ISHAM 2024 clinical state**	**Historical analogue**	**Operational definition**	**Clinical use/management**
Acute ABPA—newly diagnosed	Stage I (acute)	Meets diagnostic criteria with active clinico-radiologic disease.	Treat unless asymptomatic; select prednisolone or itraconazole by severity and toxicity risk.
Acute ABPA—exacerbation	Stage III (exacerbation)	Sustained clinical worsening for ≥14 days or radiologic worsening, plus total IgE ≥50% above the stable new baseline, after excluding other causes.	Treat as acute disease; combination therapy is favored for recurrent exacerbations.
Response	Early treatment response	At 8 weeks: ≥50% symptom improvement plus major radiologic improvement or ≥20% total-IgE decline.	Continue and complete the selected regimen; establish a new baseline after stability.
Remission	Stage II (remission)	Sustained clinico-radiologic improvement for ≥6 months off glucocorticoids, without a ≥50% IgE rise; remission may be maintained on biologic or long-term antifungal therapy.	Monitor; do not pursue IgE normalization.
Treatment-dependent ABPA	Stage IV (glucocorticoid-dependent)	≥2 consecutive exacerbations within 3 months after stopping glucocorticoids, or worsening during taper on two occasions.	Consider long-term azole, phenotype-directed biologic, and toxicity reduction.
Advanced ABPA	Stage V (fibrotic)	Extensive bronchiectasis (≥10 segments) with cor pulmonale or chronic type 2 respiratory failure.	Manage bronchiectasis, infection risk, oxygen/ventilation needs, and complications in specialist care.

**Table 3:** Major differential diagnoses of allergic bronchopulmonary aspergillosis.

**Disease**	**Typical Clinical Features**	**Features Distinguishing it from ABPA**
Severe asthma with fungal sensitization	Severe asthma and fungal-specific IgE	Does not meet full ISHAM ABPA criteria; no characteristic ABPA imaging pattern.
Eosinophilic pneumonia	Systemic illness, eosinophilia, peripheral opacities	No required *A. fumigatus* sensitization/IgE pattern; bronchoalveolar eosinophilia may dominate.
EGPA	Asthma, eosinophilia, sinus disease, neuropathy or vasculitic manifestations	Antineutrophil Cytoplasmic Antibody (ANCA) may be present; extrapulmonary vasculitis is not typical of ABPA.
CPA	Chronic symptoms, cavities, fungal ball, pleural thickening	Cavitary structural disease and microbiologic/serologic evidence of chronic infection rather than allergic mucus plugging.
Non-ABPA bronchiectasis	Chronic cough, sputum and recurrent infection	Evaluate causes including Primary Ciliary Dyskinesia (PCD); lacks mandatory fungal sensitization/IgE pattern.
ABPM	ABPA-like syndrome driven by a non-*Aspergillus* fungus	Repeated culture of the causative fungus and organism-specific sensitization; *Aspergillus* tests may be negative.

**Table 4:** Treatment options, outcome metrics, and monitoring considerations in ABPA.

**Intervention**	**Usual Role**	**Typical Regimen**	**Key evidence/outcomes**	**Monitoring and Cautions**
Prednisolone	Acute ABPA when rapid control is required	0.5 mg/kg/day for 2–4 weeks, then taper; total course about 4 months	Higher 6-week composite response than itraconazole (100% *vs* 88%) in one unblinded RCT.	Hyperglycemia, infection, weight, blood pressure, adrenal and bone toxicity.
Itraconazole	Alternative monotherapy for selected acute disease; steroid-sparing therapy	Conventional capsules 400 mg/day or super-bioavailable formulation per guideline for about 4 months	Similar longer-term IgE and exacerbation outcomes to prednisolone in the 2018 trial; older placebo-controlled evidence supports steroid sparing.	TDM target trough ≥0.5 mg/L; liver tests; CYP3A4 interactions; formulation/acid effects; heart failure and QT risk.
Voriconazole	Alternative if itraconazole cannot be used; not preferred first-line	200 mg twice daily, individualized	Systematic-review evidence across oral azoles (including studies of voriconazole) suggests clinical benefit, but certainty is low or very low and comparative evidence remains limited.	TDM trough ≥1 mg/L; hepatic, visual, neurologic, photosensitivity and interaction risks.
Prednisolone + itraconazole	Recurrent exacerbations	Use guideline monotherapy doses with coordinated taper/TDM	2022 RCT: 1-year exacerbation 20.6% *vs* 33.0% with prednisolone alone; *P*=0.054.	Combined toxicity; not routine for every newly diagnosed patient.
Omalizumab	Treatment-dependent allergic/high-IgE phenotype	Licensed severe-asthma dosing table by weight and pretreatment IgE	Meta-analytic observational signal: −2.29 exacerbations/year, −10.91 mg/day oral corticosteroid, +10.09 percentage points FEV_1_.	ABPA use is off-label; table limits vary by region; no validated high-dose ABPA strategy.
Mepolizumab / benralizumab	Treatment-dependent eosinophilic phenotype, especially mucus plugging	Licensed severe-asthma dosing	Retrospective n=29 series: fewer exacerbations and oral corticosteroids; mucus plugs improved in 82%.	Observational ABPA evidence; interpret IgE cautiously because clinical response can occur without a fall.
Dupilumab	Treatment-dependent broad type 2 phenotype or IgE outside omalizumab table	Licensed severe-asthma dosing	Case-level/cohort signals; completed phase 2 trial registry exists, but no head-to-head superiority evidence.	Transient eosinophilia, conjunctivitis; ABPA use is off-label.

**Table 5:** Practical monitoring framework for allergic bronchopulmonary aspergillosis.

**Parameter**	**Suggested Timing**	**Interpretation/action**
Symptoms and exacerbations	At every review; 8–12 weeks after starting acute therapy	Response includes ≥50% symptom improvement. Reassess alternative causes of deterioration before labeling relapse.
Total IgE	At 8–12 weeks; every 3–6 months in year 1, then every 6–12 months	A ≥20% fall supports response. A ≥50% rise from the stable new baseline is required, with clinical/radiologic worsening, for exacerbation. Do not pursue normalization.
Spirometry (FEV_1_)	At response assessment and follow-up; more often if unstable	A meaningful decline supports reassessment but is not specific for ABPA.
Chest imaging	Radiograph at response assessment; CT at baseline and for unexplained deterioration	Assess infiltrates, collapse, mucus plugging, HAM, and bronchiectasis progression; avoid routine serial CT when stable.
Blood eosinophils	Baseline and when the phenotype or competing diagnosis is uncertain	Useful for diagnosis/phenotyping; treatment and biologics can suppress counts.
*A. fumigatus*-specific IgE/IgG	At diagnosis; not routinely serial for response	Do not use isolated changes to define remission or relapse.
Liver tests and interactions	Before azole; periodically during therapy and after medication changes	Check aspartate aminotransferase (AST), alanine aminotransferase (ALT), bilirubin, symptoms, and CYP3A4 interactions; act according to severity and Upper Limit of Normal (ULN).
Azole TDM	About 2 weeks and 3 months; after dose/formulation change, failure, toxicity, or interaction	Itraconazole trough ≥0.5 mg/L; voriconazole trough ≥1 mg/L. Correct administration/interactions before dose escalation and repeat after adjustment.
Glucocorticoid toxicity	Baseline risk assessment and during prolonged/repeated therapy	Monitor glucose, blood pressure, weight, infection, adrenal and bone risk; consider dual-energy X-ray absorptiometry (DXA) and preventive therapy according to standard osteoporosis guidance.

## LIST OF ABBREVIATIONS

ABPA: Allergic bronchopulmonary aspergillosis
ABPM: Allergic bronchopulmonary mycosis
ALT: Alanine aminotransferase
ANCA: Antineutrophil cytoplasmic antibody
AST: Aspartate aminotransferase
BAT: Basophil activation test
CF: Cystic fibrosis
CFTR: Cystic fibrosis transmembrane conductance regulator
COPD: Chronic obstructive pulmonary disease
CPA: Chronic pulmonary aspergillosis
CYP3A4: Cytochrome P450 3A4
DC: Dendritic cell
DXA: Dual-energy X-ray absorptiometry
EGPA: Eosinophilic granulomatosis with polyangiitis
EQUAL: European Confederation of Medical Mycology Quality of Clinical Management
FeNO: Fractional exhaled nitric oxide
FEV_1_: Forced expiratory volume in 1 second
HAM: High-attenuation mucus
HLA: Human leukocyte antigen
HRCT: High-resolution computed tomography
IDSA: Infectious Diseases Society of America
Ig: Immunoglobulin
ILC2: Type 2 innate lymphoid cell
IL: Interleukin
ISHAM: International Society for Human and Animal Mycology
LFT: Liver function test
PCD: Primary ciliary dyskinesia
RCT: Randomized controlled trial
SAFS: Severe asthma with fungal sensitization
TDM: Therapeutic drug monitoring
Th2: Type 2 T-helper cell
TLR: Toll-like receptor
TSLP: Thymic stromal lymphopoietin
ULN: Upper limit of normal
